# Automatic Detection of Focal Cortical Dysplasia Type II in MRI: Is the Application of Surface-Based Morphometry and Machine Learning Promising?

**DOI:** 10.3389/fnhum.2021.608285

**Published:** 2021-02-19

**Authors:** Zohreh Ganji, Mohsen Aghaee Hakak, Seyed Amir Zamanpour, Hoda Zare

**Affiliations:** ^1^Medical Physics Research Center, Mashhad University of Medical Sciences, Mashhad, Iran; ^2^Epilepsy Monitoring Unit, Research and Education Department, Razavi Hospital, Mashhad, Iran; ^3^Department of Medical Physics, Faculty of Medicine, Mashhad University of Medical Sciences, Mashhad, Iran

**Keywords:** epilepsy, focal cortical dysplasia, image processing, machine learning, computer-aided diagnosis

## Abstract

**Background and Objectives:**

Focal cortical dysplasia (FCD) is a type of malformations of cortical development and one of the leading causes of drug-resistant epilepsy. Postoperative results improve the diagnosis of lesions on structural MRIs. Advances in quantitative algorithms have increased the identification of FCD lesions. However, due to significant differences in size, shape, and location of the lesion in different patients and a big deal of time for the objective diagnosis of lesion as well as the dependence of individual interpretation, sensitive approaches are required to address the challenge of lesion diagnosis. In this research, a FCD computer-aided diagnostic system to improve existing methods is presented.

**Methods:**

Magnetic resonance imaging (MRI) data were collected from 58 participants (30 with histologically confirmed FCD type II and 28 without a record of any neurological prognosis). Morphological and intensity-based features were calculated for each cortical surface and inserted into an artificial neural network. Statistical examinations evaluated classifier efficiency.

**Results:**

Neural network evaluation metrics—sensitivity, specificity, and accuracy—were 96.7, 100, and 98.6%, respectively. Furthermore, the accuracy of the classifier for the detection of the lobe and hemisphere of the brain, where the FCD lesion is located, was 84.2 and 77.3%, respectively.

**Conclusion:**

Analyzing surface-based features by automated machine learning can give a quantitative and objective diagnosis of FCD lesions in presurgical assessment and improve postsurgical outcomes.

## Introduction

Focal cortical dysplasia (FCD) is inherently epileptogenic and can be an essential reason behind refractory epilepsy (Fauser et al., 2015). FCD, described initially by Taylor et al. (1971), was first considered a rare kind of cortical development malformations. Using the advent of contemporary imaging techniques and histopathological inspections, the rate of FCD in the epileptic population seemed to be higher than initially expected, but despite the advancement of these methods, diagnosing small and subtle lesions is still challenging (Becker, 2006; Harvey et al., 2008), and the results of examining images of these lesions are often normal [“magnetic resonance imaging (MRI)-negative”] (Ahmed et al., 2015). Furthermore, FCD is not a uniform disorder and carries a wide variety of sufferers with clinical and radiological reports from minor epileptic seizures in adolescence with dubious symptoms of a lesion on MRI to more severe epileptic seizures in childhood with apparent MRI signs (Krsek et al., 2009; Lerner et al., 2009). For patients with FCD, epilepsy surgery may be a treatment option. The total removal of FCD lesions results in 50–75% improvement in patients (Cossu et al., 2008; Krsek et al., 2009; Chen et al., 2014; Choi et al., 2018). Therefore, the diagnosis of these lesions is critical in preoperative MRI because the postoperative outcome in patients with positive MRI improves significantly (Berg et al., 2003; Bien et al., 2009). Moreover, FCD is the most typical pathology among patients with MRI-negative epilepsy (Wang et al., 2013a). FCD histology is dependent on the lamination changes (type I), dysplastic neurons, and balloon cells (type II) (Blu et al., 2011). The MRI features of FCD include focal cortical thickening, decreased cortical T1 intensity, increased T2 signal, and gray–white matter blurring (Colombo et al., 2009, 2012; Leach et al., 2014). In T1-weighted scans, cortical thickness malformations, blurring of gray matter–white matter (GM–WM) boundary, and abnormal sulcus structure are FCD lesion features, and in T2/fluid-attenuated inversion recovery (FLAIR) images, signal hyperintensity is seen in FCD and transmantle sign lesions, particularly in FCD IIb (Barkovich et al., 1997; Wang et al., 2013b). MR images of patients with FCD show in 60% of the cases gray matter thickness increasing, 74% GM–WM interface blurring, 63% white matter signal hyperintensity, 19% structural atrophy, and 34% other signal changes due to the transmantle sign (Colombo et al., 2003; Lerner et al., 2009). Sometimes, these features occur together and make the lesion more visible. A combination of cortical thickness increasing, GM–WM junction blurring, and transmantle sign was seen in 64% of patients with FCD II (Mellerio et al., 2012). The challenge in many patients is to determine the exact location of the lesion tissue. Some FCDs are detected in conventional neuroimaging, and the identification of other types which are quite subtle requires advanced imaging and computational techniques (Rosenow and Lüders, 2001; Harvey et al., 2015). Moreover, the visual interpretation of MRI is very subjective and depends on the radiologist’s skills (Kassubek et al., 2002; Wilke et al., 2003; Colliot et al., 2006). Therefore, there is a pressing need to explore objective methods which can help accurately identify FCD in MRI. Quantitative postprocessing approaches were designed to solve some of the visual detection issues of FCDs with MRI (Kassubek et al., 2002). Many studies have examined the morphometric features of MRI in patients with FCD. Martin et al. (2017) compared four voxel-based morphometric methods. They examined four VBM methods: three T1 image-based methods (gray matter volume, gray matter concentration, and white–gray matter junction map) and one FLAIR-based method to evaluate its ability to detect FCD lesions with positive MRI and negative MRI. Wong-Kisiel et al. (2018) used morphometric analysis program (MAP) as a complement to visual MRI analysis in the diagnosis of FCD. Gill et al. (2017) proposed an automatic FCD lesion detection algorithm using surface-based and intensity-based features in T1, FLAIR, and FLAIR/T1 images. We designed and implemented a computer-aided diagnostic system to identify FCD lesions, assuming that morphometric and machine learning methods can accurately detect and locate FCD lesions. Firstly, we acquired structural images (T1 and T2-FLAIR sequences), performed image preprocessing (cortical reconstruction), and extracted surface features (morphological and intensity-based) from each Desikan–Killiany atlas (Desikan et al., 2006) region (34 in each hemisphere). Then, by performing machine learning models to image classification and selecting the artificial neural network as the appropriate algorithm, this method was used to detect normal images from FCD lesions as well as lesion location.

## Materials and Methods

### Ethics

This study involving human participants were reviewed and approved by the Research Ethics Committee of the School of Medicine, Mashhad University of Medical Sciences. Since we used pre-prepared data, there is no need for consent statement (Ethics code: IR.MUMS.fm.REC.1396.506).

### Participants

Thirty individuals with verified FCD type II were involved in the present research. To determine specificity, we also included a control group of 28 adults without a record of any neurological prognosis from the ADNI (adni.loni.ucla.edu) database. All individuals were right-handed. The patients and control demographics and lesion characteristics are summarized in Table 1. As shown in Table 1, 20 patients had positive MRI and 10 patients had negative MRI. This means that in positive MRI, the lesions can be identified visually (although hard), but in negative MRI, the lesions are not visually detectable. In this study, lesions were detected in MRI-negative cases using PET imaging modality.

**TABLE 1:** The demographic information of patients and controls.

	Total patients = 30		
	MRI-negative = 10	MRI-positive = 20	Normal database = 28
M/F	5/5	10/10	13/15
Age at onset (years)	15.4 (0.18–30)	13.3 (0.5–27)	–
Age at MRI scan (years)	25.9 (1–46)	21.6 (1–38)	30.1 (5–46)
MRI scanners	Philips	Philips	Siemens

### MR Imaging

We used two different scanners with two magnetic fields: 1.5 and 3 T. According to Jin et al. (2018), the type of scanners does not affect the classification performance in FCD patients. Regarding the difference of magnetic fields, according to the study of Mellerio et al. (2014) which evaluated the difference between two scanners 1.5 and 3 T, it was observed that an excellent spatial resolution for three-dimensional (3D) T1 sequences of 1.5 T images is also available, due to the limited difference between two magnetic fields for the detection of cortical features. In this study, the focus was on the cortical features (thickness, blurring, and curvature). For the control subjects from the ADNI database, 21 subject images were scanned on a Siemens (Munich, Germany) 3.0 T scanner: 3D T1-MPRAGE sequence [repetition time (TR) = 2,300 ms, echo time (TE) = 2.98 ms, slice thickness = 1 mm, no gap] and 3D T2-FLAIR (TR = 4,800 ms, TE = 343 ms, slice thickness = 1 mm, no gap), and seven subjects underwent scanning using the Siemens 1.5 T scanner: 3D T1-MPRAGE sequence (TR = 2,300 ms, TE = 3.05 ms, slice thickness = 1.2 mm, no gap) and 3D T2-FLAIR (TR = 6,000 ms, TE = 418 ms, slice thickness = 0.9 mm, no gap).

The patients’ MRI datasets have been obtained with Achieva^®^ 1.5 T magnet (Philips Healthcare; Best, Netherlands): 3D volume fast field echo (FFE) T1-weighted sequence (TR = 7.3 ms, TE = 3.3 ms, slice thickness = 0.9 mm, no gap) and 3D T2-FLAIR (TR = 140 ms, TE = 11 ms, slice thickness = 0.9 mm, 0.6 mm interslice gap).

### Method Selection

In this study, which is an extract from a dissertation (Mashhad University of Medical Sciences, 2019), we used the best method for each part of the process and analysis: (1) In the preprocessing and skull stripping stage, we compared the three methods of automatic skull removal with manual skull removal (gold standard) and selected the best method. (2) For the image processing and segmentation section, we compared three automatic methods with the manual segmentation (gold standard) performed by three experienced radiologists (a very hard and time-consuming task), and for the essential tissue segmentation for FCD lesion detection, i.e., WM and GM, the FreeSurfer software was selected as a better and more efficient method, and after processing, all segments were visually checked by a radiologist. (3) For the selection of the classification method, after training, three widely used classifiers, namely the decision tree, support vector machine, and artificial neural network, were employed, and after evaluating the results, the best one was selected which was the artificial neural network. Regarding the decision tree and SVM classifications, the images were examined using different *K*-folds, and in each *K*, the machine trained 30 times, and the average results were considered. For the artificial neural network method, the classifier was trained 30 times, and the average of evaluations was obtained. Finally, we report the best results in this article. The evaluation results of the other methods are awaiting acceptance in another article.

### Cortical Reconstruction

The FreeSurfer software (version 6.0; Athinoula A. Martinos Center for Biomedical Imaging at Massachusetts General Hospital, Boston, MA, United States) (Dale, 1999; Dale et al., 1999; Fischl and Dale, 2000) was used to apply cortical reconstruction by the recon-all pipeline (Fischl et al., 1999). The steps of this reconstruction included the following: (1) the transfer of raw image data voxels to isotropic space, (2) image normalization for bias field correction, (3) skull removal, (4) the stages of automatic subcortical segmentation, (5) white matter segmentation, and (6) tessellation of the gray matter–white matter interface. In addition to the mentioned steps, another stage was performed to improve the pial surfaces using FLAIR images and different contrasts in these images.

### Manual Lesion Segmentation

**FIGURE 1:**
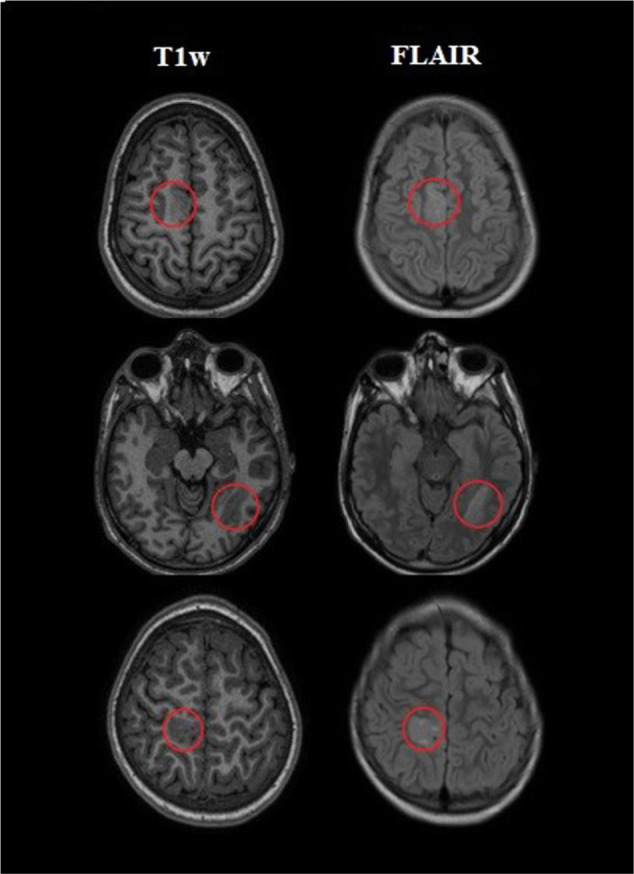
Examples of three patients with a labeled lesion. The first column on the left indicated the presurgical three-dimensional T1-weighted (T1w) images which were utilized as input for the processing. The next column revealed T2-weighted fluid-attenuated inversion recovery (FLAIR) images on the same slice.

Two experienced neurologists segmented FCD lesions using the ITK-SNAP software [version 3.6.0; Penn Image Computing and Science Laboratory (PICSL) and Scientific Computing and Imaging Institute (SCI), United States]. Thus, lesion tissue was labeled as 1 and healthy tissue as 0. Figure 1 shows examples of segmented lesions. Manual lesion segmentation was used as the ground truth for the performance analysis and to validate the classification results in lesion localization.

### Feature Extraction

We extracted morphological and intensity-based features. According to the Desikan–Killiany atlas, the cerebral cortex was parcellated into 34 regions (ROIs) per hemisphere. In general, for each ROI, the average cortical thickness, Gaussian curvature, mean curvature, intrinsic curvature index, and folding index; statistical indices including mean, min, max, range, and standard deviation; and SNR for intensity contrast were extracted. Table 2 shows the morphological features extracted from the left hemisphere of a normal MRI.

**TABLE 2:** Morphological features extracted from the left hemisphere of normal MR image, code H_0416.

StructName	ThickAvg	ThickStd	MeanCurv	GausCurv	FoldInd	CurvInd
bankssts	2.845	0.461	0.098	0.032	10	1.6
caudalanteriorcingulate	1.934	0.842	0.137	0.042	13	1
caudalmiddlefrontal	2.563	0.457	0.116	0.04	39	4.3
cuneus	2.142	0.414	0.128	0.036	45	4.7
entorhinal	2.507	1.146	0.147	0.05	7	1
fusiform	2.586	0.818	0.137	0.046	79	7.9
inferiorparietal	2.546	0.537	0.136	0.044	98	10.1
inferiortemporal	2.564	0.866	0.147	0.047	84	7.7
isthmuscingulate	2.332	0.871	0.118	0.035	18	1.7
lateraloccipital	2.362	0.447	0.128	0.034	104	10.1
lateralorbitofrontal	2.699	0.69	0.143	0.056	63	7.3
lingual	2.217	0.515	0.138	0.045	71	7.8
medialorbitofrontal	2.336	0.891	0.147	0.062	58	5.9
middletemporal	2.715	0.892	0.134	0.042	62	6.2
parahippocampal	1.939	0.621	0.096	0.021	6	0.8
paracentral	2.617	0.5	0.108	0.045	39	4.9
parsorbitalis	2.938	0.507	0.174	0.056	20	2
pericalcarine	1.969	0.442	0.125	0.03	22	2.8
posteriorcingulate	2.254	0.831	0.128	0.038	21	1.7
precentral	2.627	0.539	0.117	0.05	167	17.4
precuneus	2.481	0.499	0.132	0.044	74	8.3
rostralanteriorcingulate	2.28	0.972	0.149	0.08	55	3.5
rostralmiddlefrontal	2.59	0.543	0.147	0.055	125	15.5
superiorfrontal	2.732	0.567	0.137	0.057	271	24.6
superiorparietal	2.358	0.458	0.118	0.031	83	8.1
superiortemporal	2.505	0.721	0.114	0.037	75	8.3
supramarginal	2.634	0.518	0.139	0.047	82	8.6
frontalpole	3.232	0.701	0.175	0.08	12	1.2
temporalpole	3.674	0.876	0.155	0.073	14	1.6
transversetemporal	2.579	0.354	0.113	0.049	13	1.8
insula	3.005	0.883	0.144	0.07	58	10.5

#### Morphological Features

The cortical thickness will be computed as the distance from the WM surface (WM–GM boundary) to the nearest possible point over the pial surface (GM–CSF boundary) (Dale et al., 1999; Han et al., 2006). The Gaussian curvature, the mean curvature, and the new features of this study including the intrinsic curvature index and the folding index, which are the geometric indices of the principal cortical surface curvature (Van Essen and Drury, 1997; Pienaar et al., 2008; Ronan et al., 2011), were calculated as follows (in these calculations, *k*_1_ and *k*_2_ are the principal curvatures at points 1 and 2 on the cortical surface): mean curvature = $\frac{1}{2}\left(k_{1}+k_{2}\right)$, Gaussian curvature = *k*_1_.*k*_2_, intrinsic curvature index is computed as |*k*_1_*k*_2_|, and folding index = |*k*_1_| (|*k*_1_|−| *k*_2_|) (Jin et al., 2018).

#### Intensity-Based Features

The intensity contrast is calculated as the ratio of GM signal intensity to WM signal, where the GM signal intensity, obtained at 30% of the cortical thickness above the GM–WM boundary and WM signal intensity, is 1 mm below the GM–WM boundary (Salat et al., 2009). Lesions with GM–WM boundary blurring compared with the healthy cortex appear to have low-intensity contrast of the GM–WM (Blumcke et al., 2017). In summary, 408 morphometric and 408 intensity-based measurements covering the entire brain per participant were used for subsequent analyses. All features were normalized using *z*-score (McLeod, 2019).

### Statistical Analysis

#### Machine Learning Classification

The images are classified for two purposes: First, the diagnosis of lesions versus nonlesional tissue was performed using the ANN classification. The second was the detection of the lesion area. To do this, the automated classification was performed in two steps. We applied ANN to detect (i) the lesional lobe of the brain and (ii) the lesional hemisphere of the brain.

##### ANN classification

Automatic FCD lesion diagnosis was performed utilizing an artificial neural network classifier carried out in MATLAB R2014 (MathWorks, Natick, MA, United States). The ANN classifier was trained with extracted features. We used the feedforward neural network to train information. It had input, hidden, and output layers. In this network, the information move only from one direction to the next. The flow of information is done by input nodes (neurons), and if there are hidden layers, they pass through and enter the output nodes (Haykin, 1994). Each node took a differently weighted combination of features. Then, the outputs were combined to determine that the features of a vertex are similar to a healthy (output value is zero) or lesional tissue (output value is one) of the cortex; 70% of the obtained data for training is dedicated. The rest was divided into two equal proportions as validation and test. Each subject in the training dataset was assigned a value of 0 (healthy cortex) or 1 (lesional cortex). Training and performance evaluations were performed with 30 iterations.

##### Lesion localization

We decided to determine the location of the lesion in two steps: first, the lesional hemisphere in two classes—right hemisphere (RH) and left hemispheres (LH); and second, the lesional lobe in four classes: frontal, temporal, parietal, and occipital.

#### Evaluation of Surface-Based Features

**FIGURE 2:**
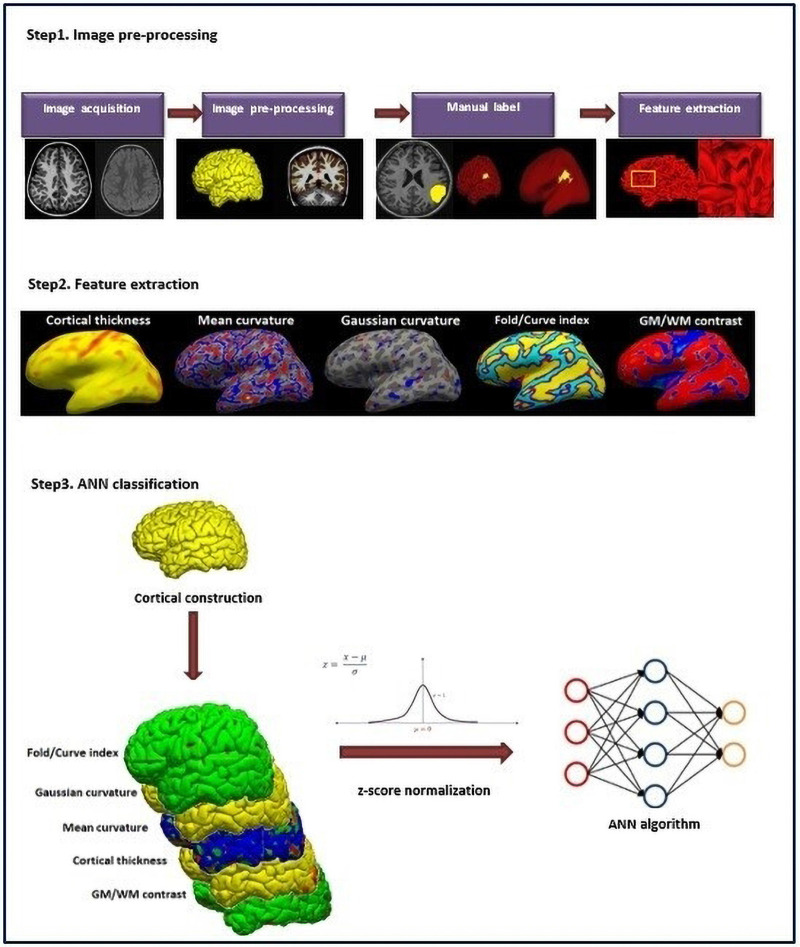
Overall procedure.

The classification performance was assessed using statistical parameters of sensitivity, specificity, and accuracy to identify the patients from the control. The accuracy and sensitivity parameters were also used to evaluate the location of the lesion. The overall procedure is shown in Figure 2.

## Results

### Demographics

Thirty clients with a radiological sign of FCD type II (10 and 20 patients with negative and positive MRI, respectively) and 28 healthy controls were involved. Due to radiological reports, lesion focus was present on the left hemisphere in 10 patients and on the right hemisphere in 20 other patients. The lesion location was labeled by an experienced neurologist: frontal lobe (*n* = 11), temporal lobe (*n* = 6), parietal lobe (*n* = 6), occipital lobe (*n* = 2), and multilobar (*n* = 5). The multilobar lesions are all located in the right hemisphere.

### Evaluation of the ANN Algorithm for the Classification of FCD Lesion and Healthy Subjects

Image classification with ANN includes three stages: training, testing, and validation. To perform these steps, we considered 48 images, consisting of 20 images of patients with positive MRI and 28 controls; 70% of these images were used for training, 15% for testing, and 15% for validation. The results show the three parameters of sensitivity, specificity, and accuracy. The mean values of these three metrics after 30 iterations were 96.7, 100, and 98.6%, respectively. Ten patients with negative MRI were used for the final validation of the classification system. After applying the ANN classification to 48 images in both normal and lesion classes, the feature vector of these patients in the lesion class was included in the classification system; consequently, the results showed 91.3% classification accuracy. The mean and standard deviation of the classifier performance are shown in Table 3 after 30 iterations.

**TABLE 3:** Mean and standard deviation of sensitivity, specificity, and accuracy parameters in the ANN classifier.

MRI	Statistic	Sensitivity	Specificity	Accuracy
MRI-positive	Mean	96.7%	100%	98.6%
	Std*	7.9	0	3.2
MRI-negative	Mean	91.3%	–	91.3%
	Std	1.9	–	1.9

*Std, standard deviation.

### The Assessment of Classifier for the Detection of Lesion Location

The ANN algorithm was first designed to identify the lesional hemisphere: the LH and the RH. Also, it was designed to detect lesional lobe into four classes: frontal, temporal, parietal, and occipital lobes. The classification accuracy in the diagnosis of the lesion area was 84.2% for lesional hemisphere and 77.3% for the lesional lobe.

## Discussion

Focal cortical dysplasia is among the most important factors behind drug-resistant epilepsy, which is treatable by surgery. The entire resection and excision of dysplastic and extra epileptogenic tissue are believed to be an essential factor in treating this lesion. However, the detection of lesions is still a challenge despite the most enhanced imaging methods. Interpreting images is a time-consuming and individual-dependent process which increases the possibility of incorrect diagnosis of the lesion. Therefore, a computer-aided diagnosis tool is needed to accurately identify lesions and restrict the region of surgery to the lesion tissue. In recent years, researches on FCD computer-aided diagnostic systems were significantly increased to assist neurologists. The present study aimed to employ quantitative surface features of MR images and machine learning techniques for the automatic identification of FCD lesions and the involved areas. Although this idea was stated in several studies in the past, there are some innovations in the present study. First, to improve the segmentation of brain images in the FreeSurfer software, the FLAIR images were applied. Second, the new two features, the fold index and the curve index, were employed, which have not been used before. The third most important which is to localize the lesions was performed in two stages: lesional hemispheres and lesional lobes, which will lead to a significant reduction in the time of FCD lesion identification.

A new computer-aided diagnostic system of FCD lesions was investigated to increase lesion detection accuracy. The designed system was capable of detecting FCD lesion as well as the lesion area. Moreover, it enabled quantitative and objective assessment of lesions against individual clinical diagnosis. The ANN classifier performance was evaluated using conventional statistical methods and quantitative analysis. The present study included 58 MRI data in neural network classifier training. In MRI-positive cases, the sensitivity, specificity, and accuracy of the classifier were 96.7, 100, and 98.6%, respectively. Previous studies have used various morphometric methods to identify FCD lesions in MRI-positive cases. In the study of Wong-Kisiel et al. (2018), the MRIs of patients with FCD were evaluated to determine the role of MAP postprocessing in brain lesion detection. Using abnormal GM extension (MAP-E) and WM and GM junction blurring in 39 patients, the sensitivity and specificity were 64 and 96% for MAP-E and 74 and 94% for MAP-J, respectively. In the study by Ahmed et al. (2015) using a surface-based method, 86% MRI-positive patients with confirmed FCD were correctly and automatically identified.

Besides, about 33% of clients in our research were MRI-negative by primary visual examination. After classifier training using FCD type II lesions with positive MRI, the classification test results with negative MRI showed high accuracy (91.3%). In addition to MRI-positive patients, in the study of Ahmed et al. (2015), patients with negative MRI were evaluated and 58% of FCD lesions were correctly identified. In the study of El Tahry et al. (2020) and Haykin (1994), 21 patients with FCD and negative MRI were evaluated by postprocessing MAP. The MAP results led to the detection of six FCD lesions (29%). Therefore, our data showed that using MRI-positive patients in training, which have initially normal MRI, have a diagnostic benefit for lesions. Mo et al. (2019) used features such as morphology, intensity, and function and designed an objective and intelligent diagnosis of FCD lesions in presurgery evaluations. The difference between their study and our study is the use of multimodal surface features. In addition to MR images, they also used PET images. The accuracy of the classification based on multimodal features was 75% for detecting FCD II lesions.

In evaluating the lesion’s location, the accuracy of the classifier for the detection of the lesional hemisphere and lesional lobe was 84.2 and 77.3%, respectively. In past works related to the localization of the lesion, Hong et al. (2014) employed another surface-based approach and reported high sensitivity (74%) in the intelligent classification of FCD type II lesions. No lesions were identified in the control group indicating excellent specificity. Adler et al. (2017) studied surface-based morphometry and neural network methods and noted a successful diagnosis rate of 73% in 22 patients which had a radiological analysis of FCD; however, the specificity had not been examined. Feng et al. (2020) defined a mean cortical thickness map for MRIs in FCD patients. They compared the location of the lesions in patients with the control group and three out of four lesion areas were detected. However, their method is not effective for lesions situated in the temporal lobe.

For the automatic identification of FCD lesions and brain regions involved, using surface features including the morphology and intensity-based features as well as quantitative methods of machine learning, a computer-aided diagnostic system for FCD lesions was presented. In MRI-positive cases, our methods showed a high sensitivity of 96.7% in patients with FCD type II and also had excellent specificity (100%) and reliable accuracy (98.6%). This method had also high accuracy to detect lesion location in the brain (84.2% in hemisphere diagnosis and 77.3% in lobe diagnosis), but similar studies have not been performed for comparison. Also, in MRI-negative cases, the results showed 91.3% accuracy.

Various classification methods were proposed in studies to detect FCD lesions. The results obtained from similar studies and the results of this research are shown in Table 4.

**TABLE 4:** Comparison of classification results in this study with other studies.

Classifier	Sensitivity (%)	Specificity (%)	Accuracy (%)
SVM (El Azami et al., 2016)	77	–	–
Decision tree (Harvey et al., 2015)	83	92	–
ANN (Besson et al., 2008)	89	–	–
ANN (Adler et al., 2017)	73	–	–
ANN (Jin et al., 2018)	73.7	90	–
ANN (this study)	96.7	100	98.6

In general, our results suggest that a completely automated machine learning method can provide a major FCD diagnosis outcome in presurgical assessment for pharmacoresistant patients. The clinical application of these results can be found in the fact that the main goal of neurologists is accurate surgery of FCD lesions, and as has been mentioned, the visual detection of epileptogenic focal is a difficult and time-consuming task. In an automatic lesion detection system using MR images, noninvasive methods can be used to assist neuroradiologists, and the costs incurred for using other methods by many patients, such as PET imaging, can be reduced.

## Conclusion

We tried to prove the importance of surface-based MRI morphometry by machine learning using a group of patients with type II FCD. This technique can be a valuable tool to boost patients’ preoperative analysis with drug-resistant epilepsy. The limitation of our study was insufficient data. As a suggestion for future studies by the increasing amount of data, the results will be improved to detect the lesion area, and other methods may also be used to identify the lesion area involved.

## Data Availability Statement

The original contributions presented in the study are included in the article/supplementary material, further inquiries can be directed to the corresponding author/s.

## Ethics Statement

This study involving human participants were reviewed and approved by Research Ethics Committee of School of Medicine-Mashhad University of Medical Sciences. Written informed consent from the participants’ legal guardian/next of kin was not required to participate in this study in accordance with the national legislation and the institutional requirements.

## Author Contributions

All authors listed have made substantial, direct and intellectual contribution to the work, and approved it for publication.

## Conflict of Interest

The authors declare that the research was conducted in the absence of any commercial or financial relationships that could be construed as a potential conflict of interest.
